# Intestinal Endometriosis in a 38-Year-Old Woman

**DOI:** 10.3390/diagnostics16152470

**Published:** 2026-08-05

**Authors:** Ruoxi Zhang, Xiaoqing Li, Aiming Yang, Qiang Wang

**Affiliations:** Department of Gastroenterology, Peking Union Medical College Hospital, Chinese Academy of Medical Sciences and Peking Union Medical College, Beijing 100730, China

**Keywords:** intestinal endometriosis, lymphatic-dilatation-like change, endoscopic recognition

## Abstract

Endometriosis is a common gynecological disorder that can involve the intestinal tract and may present with bleeding, bowel obstruction, and, rarely, perforation or malignant transformation. Intestinal endometriosis can mimic malignant tumors or other conditions, which may lead to unnecessary aggressive surgical resections. It continues to be a challenging diagnosis to make preoperatively. We report a case of intestinal endometriosis with endoscopic features of lymphatic dilatation-like changes, which correlated with the pathological findings, aiming to provide a reference for the endoscopic recognition of intestinal endometriosis.

**Figure 1:**
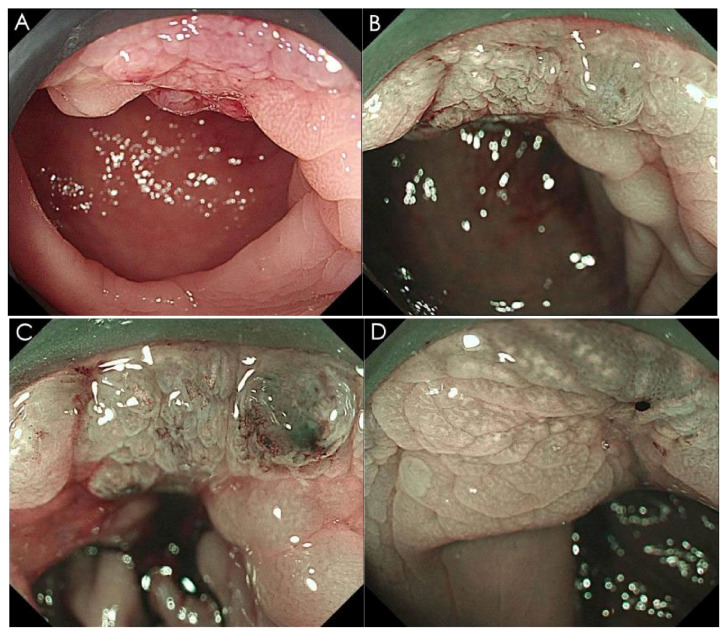
Endoscopic images of intestinal endometriosis. A 38-year-old woman presented with a 3-month history of cyclic hematochezia, which was strictly confined to the menstrual period and occurred with each cycle. Her menstrual cycles had previously been regular (28 days) with moderate flow, but she reported persistent dyspareunia and worsening dysmenorrhea (NRS from 4 to 7) during the three months. She denied anemia, dyschezia, or chronic pelvic pain, and had never sought medical evaluation or treatment for these complaints. Her obstetric history was gravida 1, para 1, with no history of infertility or prior surgery. CEA and CA19-9 were within normal ranges. Using a standardized protocol for bowel nodule assessment, combined transrectal and transvaginal ultrasonography revealed a hypoechoic lesion at the anterior rectal wall, 8–10 cm from the anal verge, measuring 15.7 × 10.4 mm in cross-section (axial plane at maximal thickness), with a longitudinal extent of 41.5 mm (sagittal plane) and circumferential involvement of 13.9% (ratio of lesion extent to total rectal circumference). The lesion was contiguous with the posterior uterine wall and involved the perirectal fat, serosa, muscularis propria, and submucosa, without evidence of abnormally enlarged lymph nodes. The sliding sign was absent. Colonoscopy performed 3 days prior to menstruation identified two discoid elevated lesions with surface nodularity (**A**), measuring approximately 20 mm and 15 mm in diameter at 10 cm and 8 cm from the anal verge, respectively. Lymphatic-dilatation-like changes and distorted, mildly dilated microvessels, were observed under magnifying endoscopy with narrow-band imaging (ME-NBI, (**B**–**D**)). Endoscopic ultrasound (EUS) demonstrated a focal hypoechoic lesion with ill-defined borders and indistinct demarcation between the mucosa and submucosa.

**Figure 2:**
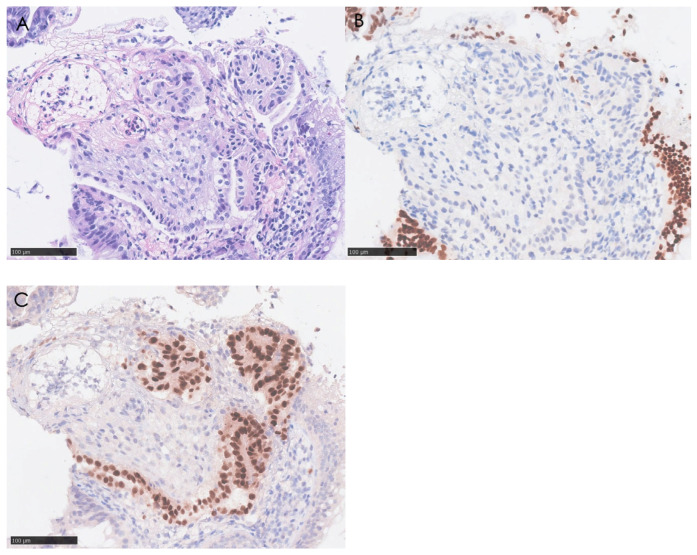
Pathology of intestinal endometriosis. Intestinal endometriosis was confirmed by four repeated bite-on-bite biopsy specimens taken from the lesion at 10 cm from the anal verge: irregular endometrial-like glands with surrounding stroma, and dilated blood and lymphatic vessels were observed on H&E staining (**A**), and immunohistochemistry demonstrated that the epithelial glands and stroma were negative for CDX2 (**B**), while the glands were positive for PAX8 (**C**). Additional CD10, ER, and PR immunostaining could not be performed because the remaining paraffin-embedded tissue was insufficient. After multidisciplinary team (MDT) discussion involving obstetrics/gynecology and general surgery, the patient elected to receive gonadotrophin-releasing hormone agonist (GnRH agonist, goserelin 3.6 mg every 28 days). After three months of hormonal therapy, the patient reported noticeable relief from dysmenorrhea, and hematochezia had completely resolved. The patient declined surgical intervention due to concerns about surgical morbidity. Intestinal endometriosis, a subtype of deep infiltrating endometriosis (DIE), accounts for 3.8–37.0% of all endometriosis cases [1], with over half of lesions predominantly affecting the rectum and sigmoid colon. Sometimes, endometriotic implants can induce secondary mucosal changes that mimic various conditions, including malignancy, inflammatory bowel disease (IBD), subepithelial lesions, and even lymphangiectasia. These conditions can be preliminarily differentiated based on their endoscopic features. Colorectal neoplasms typically present with irregular mucosal surfaces and distorted pit patterns, with irregular microvessels visible under magnifying endoscopy with ME-NBI. IBD often manifests as continuous or skip lesions with mucosal hyperemia, erosion, ulceration, and loss of vascularity, rather than localized submucosal protrusions. Lymphangioma or lymphangiectasia appears as white or yellowish elevated lesions with smooth surfaces. Other subepithelial tumors generally present as submucosal elevations with smooth overlying mucosa, and ME-NBI usually reveals normal pit patterns and microvascular architecture. Magnifying endoscopy with ME-NBI may help in their differentiation, which has been reported only in a few cases. Tomiguchi et al. reported sparsely distributed round pits within granular areas, which were presumed to indicate the openings of endometrial glands surrounded by endometrial stroma [2]. Straight microvessels among radially arranged ducts, along with an avascular area at the top of the papillary bulge, have also been described [3]. Our case further enriches the ME-NBI features of intestinal endometriosis, revealing lymphatic-dilatation-like changes and distorted, mildly dilated microvessels, which, to our knowledge, have not been emphasized in previous reports. Previous studies have shown increased lymphatic vessel density and upregulated expression of lymphangiogenic factors vascular endothelial growth factor (VEGF)-C/VEGF-D in ectopic endometrial tissues [4]. Consistent with findings in other diseases [5], the macrophage- and cytokine-mediated regulation of VEGF-C and VEGF-D [6,7,8] indicates that lymphangiogenesis forms part of the inflammatory pathology. Lymph node involvement has also been observed in patients with intestinal endometriosis [9]. These findings suggest that lymphatic vessels contribute to the pathogenesis of endometriosis. Accordingly, lymphangiectasia may represent one of the potential endoscopic features that could raise suspicion for intestinal endometriosis. These features may facilitate endoscopic recognition and support the future development of ME-NBI-guided targeted biopsy to improve preoperative diagnostic accuracy. In addition to endoscopic differentiation, histopathological biopsy is a key diagnostic tool for intestinal endometriosis, especially when malignancy must be excluded. In our case, two separate mucosal protrusions were observed at 8 cm and 10 cm from the anal verge on colonoscopy, whereas ultrasonography demonstrated that these represented two elevated aspects of a single contiguous infiltrative lesion with a longitudinal extent of 41.5 mm. Because the lesion extended into the submucosa, four repeated bite-on-bite biopsy specimens were obtained from multiple points within the lesion at 10 cm from the anal verge using standard forceps. This approach yielded diagnostic tissue despite ultrasonographic preservation of the mucosal layer. Biopsy was performed three days before menstruation, and all specimens were reviewed by a pathologist with expertise in gynaecological pathology. Immunohistochemically, ectopic endometrial glands express ER, PgR, and CK7, whereas the stroma expresses CD10, ER, and PgR. Normal rectal mucosa expresses CK20 and CDX2, but not these markers. PAX8 is a highly sensitive epithelial marker for the glandular component of extragenital endometriosis. Ref. [10], serving as a useful diagnostic tool [11,12], particularly in cases with relatively typical clinical presentations but limited tissue availability, such as the one reported here. Research on non-invasive biomarkers for endometriosis has explored a wide range of metabolic and inflammatory candidates, including the plasma and peritoneal fluid leptin/BMI ratio [13], as well as annexin A2 [14], cadherin 12 [15], metalloproteinases, growth factors, cytokines, chemokines, and microRNAs [16]. Although some of these markers may correlate with certain disease characteristics, they currently lack sufficient discriminatory value to replace imaging or histopathological assessment. Therapeutic strategies are individualized according to the patient’s fertility status, disease severity, and complications, including surgery or hormonal manipulations. The initial management of patients with mild to moderate symptoms usually involves medical therapy, such as hormonal agents and nonsteroidal anti-inflammatory drugs. Given the absence of severe complications such as intestinal obstruction or massive bleeding, and the patient’s lack of fertility requirements, GnRH agonist therapy was prioritized in accordance with the patient’s preference. GnRH agonist suppresses pituitary gonadotropin secretion, thereby inhibiting the growth of endometriotic lesions. Different surgical approaches, including shaving excision, disk resection, and segmental resection, should be tailored to lesion location while balancing the risks of disease recurrence and postoperative adverse events [1]. In conclusion, this case highlights that gastroenterologists should include endometriosis in the differential diagnosis of hematochezia in women of childbearing age, which underscores the importance of thorough history-taking. Gastroenterologists need to be familiar with its unusual endoscopic appearances and perform biopsies tailored to the specific characteristics of the disease when clinical suspicion of intestinal endometriosis is high.

## Data Availability

The raw data supporting the conclusions of this article will be made available by the authors on request.
